# Safety and Efficacy of Citrate Anticoagulation in Therapeutic Plasma Exchange: A Clinical Study

**DOI:** 10.3390/clinpract15100172

**Published:** 2025-09-23

**Authors:** Ciprian Gîndac, Tamara Mirela Poroșnicu, Nilima Rajpal Kundnani, Nicoleta Sgăvârdea, Claudiu Rafael Bârsac, Vlad Meche, Adelina Băloi, Laura Alexandra Nussbaum, Ovidiu Horea Bedreag, Dorel Săndesc, Marius Păpurică

**Affiliations:** 1 Department of Anaesthesia and Intensive Care, “Victor Babes” University of Medicine and Pharmacy, 300041 Timisoara, Romania; 2 Clinic of Anesthesia and Intensive Care, “Pius Brinzeu” Emergency Clinical County Hospital, 300723 Timisoara, Romania; 3 Compartment of Anesthesia and Intensive Care, “Dr. Victor Babes“ Infectious Diseases and Pulmonology Clinical Hospital, 300310 Timisoara, Romania; 4 University Clinic of Internal Medicine and Ambulatory Care, Prevention and Cardiovascular Recovery, Department VI-Cardiology, “Victor Babes” University of Medicine and Pharmacy, 300041 Timisoara, Romania; 5 Research Centre of Timisoara Institute of Cardiovascular Diseases, “Victor Babes” University of Medicine and Pharmacy, 300041 Timisoara, Romania; 6 Doctoral School, “Victor Babes” University of Medicine and Pharmacy, 300041 Timisoara, Romania; 7 Department of Pedopsychiatry, “Victor Babes” University of Medicine and Pharmacy, 300041 Timisoara, Romania

**Keywords:** citrate, plasma, therapeutic plasma exchange, anticoagulation, heparin

## Abstract

**Introduction:** TPE (therapeutic plasma exchange) has proven to be an extremely effective treatment for a range of conditions, especially over the past 20 years. Anticoagulation with heparin is currently the accepted recommendation for therapeutic plasma exchange sessions. However, the hypercoagulable state and hyperviscosity in some patients requiring TPE present a challenge, particularly during the first session, due to an increased risk of circuit clotting. Citrate anticoagulation has been proposed for extracorporeal therapies such as hemodiafiltration where heparin is contraindicated. Nevertheless, citrate anticoagulation is still generally avoided in patients undergoing TPE. **Materials and Methods:** A total of 26 patients underwent 52 TPE sessions using citrate. Fifteen patients received citrate from the beginning of therapy, accounting for 29 sessions, and eleven patients were switched to citrate after initially starting with heparin, when an imminent risk of circuit clotting quickly became evident—23 sessions in total. The imminent risk of circuit clotting was assessed by a continuous and accelerated increase in transmembrane pressure despite heparin anticoagulation. The effectiveness of citrate anticoagulation and its safety for patients were evaluated. **Results:** Of the 23 sessions where there was a risk of circuit clotting, citrate was added on top of heparin in those sessions; 21 sessions were successfully completed. It can be said that the kits were saved in these cases. Among the 29 TPE sessions that used citrate from the start, 27 were completed successfully, even though the patients were considered to have a hypercoagulable status. No cases of citrate toxicity were identified. **Conclusions:** TPE with citrate is a safe option for patients. It can preserve TPE kits from the beginning or during treatment in patients with hypercoagulability. Citrate can be also be used when heparin is contraindicated or ineffective.

## 1. Introduction

Plasma purification sessions have become widespread globally over the past 20 years. Among these, hemofiltration with or without an adsorber and therapeutic plasma exchange (TPE) are the most commonly used techniques [1]. TPE has proven to be an extremely effective treatment for a variety of pathologies [2]. Anticoagulation with heparin is currently the gold standard for these extracorporeal purification techniques [3]. The hypercoagulable state and hyperviscosity in some patients requiring TPE pose a problem, particularly during the first session, due to the increased risk of circuit clotting [4]. Citrate anticoagulation has been proposed for extracorporeal purification therapies such as hemodiafiltration, where heparin is contraindicated [5]. Citrate anticoagulation has proven to be significantly more effective in preventing circuit clotting [6]. However, it is still avoided in TPE sessions, and the machines used for treatments other than hemofiltration are not adapted for citrate use. In practice, circuit clotting during TPE results in premature treatment termination, failure to achieve therapeutic goals, increased patient risk due to incomplete exchange, and additional costs from wasted filters and blood products. This highlights the urgent need for safe and effective anticoagulation alternatives.

The reasons citrate is not used as the first-line anticoagulant in TPE by filtration are not clearly explained [5,7]. One possible reason may be the potential toxicity from citrate accumulation, especially since citrate is also present in the fresh frozen plasma bags used as replacement fluid. Another reason could be the higher cost of citrate anticoagulation compared to heparin [8,9].

Currently, citrate is used as a regional anticoagulant in hemofiltration sessions lasting 24–72 h [10,11]. Its use in TPE sessions, which last on average 2–4 h, should not pose an increased risk of citrate toxicity if calcium is administered in accordance with citrate levels in the blood flow, as outlined in standard protocols [12]. The citrate dose in fresh frozen plasma is 20 mmol/L, similar to the 18 mmol/L solutions most commonly used [13,14].

In Romania, therapeutic plasma exchange machines used in hospitals operate through the filtration method. Machines that perform TPE via centrifugation are an exception. For those using filtration as the method of plasmapheresis, the software does not provide the option for citrate anticoagulation, and the solution administered in predilution is not automatically removed as it is in hemofiltration procedures.

Our research aims to provide a solution for the engineers who will develop software for future TPE machines that will use citrate as an anticoagulant.

In our study, we aimed to administer citrate to two groups of patients. In the first group, citrate was used for patients who showed an obvious increased risk of kit clotting during TPE sessions. This risk was identified by closely monitoring the TMP (transmembrane pressure) curve, which rose continuously despite heparin administration, in an attempt to save the kit. The second group included patients suspected of having a hypercoagulable state or a pathology with high risk of plasma filter clotting—either the previously used kit had clotted very quickly, or the patients were not suitable for heparin use. In this second group, citrate anticoagulation was initiated from the start.

Our aim was to evaluate the safety of citrate administration in these two groups of patients, as well as the financial benefit of saving both time and money by completing a TPE session started with heparin and continued with citrate, or by conducting therapy using citrate regional anticoagulation from the outset. Our study is important because it provides real-world evidence that citrate anticoagulation can be safely applied in TPE sessions performed with filtration devices, an area with limited published data. Demonstrating its safety and effectiveness may help reduce session interruptions, optimize ICU resources, and provide an alternative when heparin is contraindicated or ineffective. The novelty of our study lies in two aspects:

- The use of transmembrane pressure (TMP) monitoring as a guide to dynamically switch from heparin to citrate to prevent imminent circuit clotting;
- The evaluation of the safety and effectiveness of citrate anticoagulation in real-world patients across multiple clinical contexts.

## 2. Materials and Methods

### 2.1. Experimental Phase, Study Design, and Setting

This was a prospective observational study conducted in the Intensive Care Units of Timisoara County Emergency Clinical Hospital and “Dr.Victor Babes” Infectious Diseases and Pulmonology Clinical Hospital, Timisoara, Romania, between January 2015 and December 2024. Over 500 TPE procedures were performed in this period.

Inclusion criteria: age ≥ 18 years, at least one TPE session, and either (a) rising TMP values ≥ 100 mmHg despite heparin anticoagulation (Group 1), or (b) known hypercoagulability or contraindication to heparin (Group 2).

Exclusion criteria: patients under 18, incomplete data, or TPE performed without monitoring of TMP or ionized calcium.

Exposure: citrate anticoagulation, either added during sessions (Group 1) or from the beginning (Group 2).

Follow-up: monitoring was performed before, during, and immediately after TPE sessions.

Data collection: demographic and clinical variables, biochemical and hemodynamic parameters, TMP trends, citrate and calcium infusion rates, session duration, and adverse events.

Between 2015 and 2025, we conducted a prospective observational study in which more than 500 TPE procedures were carried out in the intensive care units of the Timisoara County Emergency Clinical Hospital and “Victor Babes” Hospital in Timisoara, involving more than 150 patients. In these procedures—especially the initial sessions for certain pathologies (COVID-19, polyradiculoneuritis, hypertriglyceridemia, myasthenia gravis)—the rate of kit clotting was higher. COVID-19 patients were a distinct category, with over 50% of kits clotting either at the start or during the first session, even though anticoagulation with heparin had been instituted according to the hospital protocol (circuit heparinization with 5000 IU/L during priming, 20–40 IU/kg as bolus, and 10–20 IU/kg/h for maintenance).

**Figure 1:**
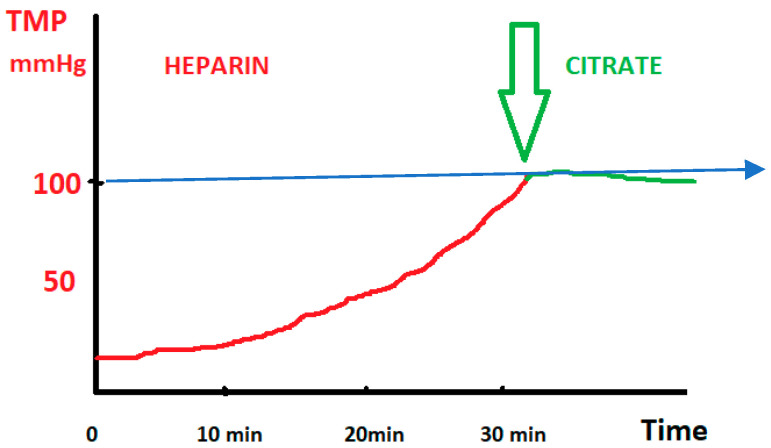
TMP curve. TMP: transmembrane pressure (measured in mmHg). Time: duration of infusion (measured in minutes). Red line shows the infusion of heparin, whereas the green line shows the introduction of citrate.

This observation led us to monitor the transmembrane pressure (TMP) curve in these patients, as a continuous rise in TMP strongly suggests an imminent risk of TPE circuit clotting, and, as a safety procedure, when the pressure reached 100 mmHg, we added citrate (Figure 1).

**Figure 2:**
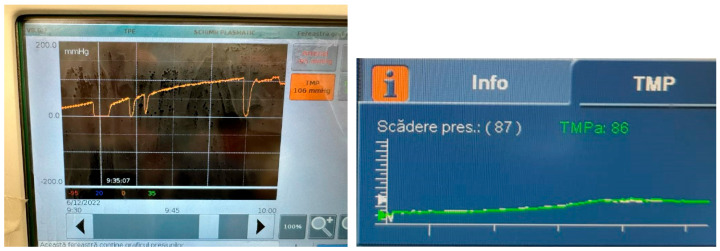
Increase in TMP (transmembrane pressure) and its attenuation through citrate administration.

When the TMP exceeds 100 mmHg, the TPE machine will trigger an alarm indicating a risk of circuit clotting. Before or at the time this alarm appeared, in a group of 23 patients, we additionally administered citrate in predilution at a rate of 2–3 mmol of citrate per liter of blood entering the filter via the blood pump (maximum 1000 mL/h of citrate solution with a concentration of 18 mmol/L) (See Figure 2).

Given that the TPE machines used in our hospitals (Prismaflex and HF440) do not have the capability to automatically eliminate this amount introduced in predilution (as is the case with hemofiltration techniques), we were required to offset this by setting a corresponding fluid loss in the patient’s prescription to avoid fluid overload. For example, if we administer 500 mL/h in predilution, we set the machine to a fluid loss of 400–500 mL/h. The amount set as replacement fluid will be lower, considering that 400–500 mL/h is already being removed. Thus, if we aim for a total replacement rate of 1200 mL/h, and we already have a 500 mL/h fluid loss, we will set an additional 700 mL/h as replacement.

In another group of patients, we initiated citrate anticoagulation from the beginning of the TPE session. These patients either had a history of rapid previous plasma filter clotting or they had a condition that presented a high risk of filter clotting during plasmapheresis.

The inclusion criteria comprised patients over 18 years of age, having undergone a TPE session, having continuous or rapidly increasing TMP (transmembrane pressure) values with a trend reaching 100 mmHg before the session’s completion, and triggering of the “filter clogged” alarm—upon which citrate regional anticoagulation was introduced in addition to heparin. These patients were considered Group 1 (23 sessions), and those for whom regional citrate anticoagulation was used from the beginning were considered Group 2 (29 sessions).

### 2.2. Standardized Protocol for Regional Citrate Anticoagulation (RCA) in TPE

Initiation triggers (any of the following): (i) continuously rising TMP with slope suggestive of imminent clotting; (ii) TMP ≥ 60–100 mmHg with an upward trend or “filter clogged” alarm; (iii) heparin contraindication or high bleeding risk; (iv) prior rapid filter clotting; (v) severe hypertriglyceridemia (>2000 mg/dL).

Citrate solution and starting rate: 0.5% sodium citrate (18 mmol/L) infused pre-filter targeting ~2–3 mmol citrate per liter of blood entering the filter. With typical blood flows of 120–130 mL/min, this corresponded to 500–800 mL/h (upper cap 1000 mL/h on Prismaflex; HF404 via external infusion pump). The exact start within this range was selected according to TMP severity.

Fluid balance coupling: Device “fluid loss” was set to 80–100% of the pre-filter citrate rate to limit net balance to ≤+60 mL/h; remaining replacement was delivered as albumin and/or FFP.

Calcium supplementation (systemic): Calcium gluconate 94 mg/mL at 0.2–0.3 mL/kg/h baseline; titrate every 30–60 min to maintain systemic iCa 1.00–1.20 mmol/L. If iCa < 0.95 mmol/L or symptoms, increase calcium by +5–10 mL/h and/or reduce citrate by 100–200 mL/h; if iCa < 0.90 mmol/L, pause citrate until iCa recovers ≥ 1.00 mmol/L, then resume at a lower rate.

Laboratory and clinical monitoring: iCa, Na^+^, K^+^, Cl^−^, pH, lactate, hemodynamics at baseline, ~90 min, and end-session; continuous ECG and non-invasive blood pressure; clinical monitoring for citrate effects (perioral/finger paresthesia, tremor, chills, arrhythmia).

Special situations: With FFP replacement (citrate load ~20 mmol/L), anticipate higher calcium requirements; prefer the lower end of citrate rates when TMP allows. For HF404 (no built-in predilution), use infusion pump pre-filter and strictly couple fluid loss to the infusion to avoid fluid overload.

De-escalation/stop rules: Reduce or stop citrate if TMP normalizes and remains stable < 60 mmHg for ≥ 30 min, or if persistent iCa instability occurs despite supplementation.

Targets: uninterrupted session completion; avoidance of symptomatic hypocalcemia or alkalosis; session duration optimization.

The protocol is also included in the Supplementary Materials section of this article, for easier access (see Supplementary Materials).

The TPE sessions were performed using the Prismaflex device (Baxter International, USA) with a TPE 2000 plasma filter, or the HF404 machine (Infomed) with a Granopen 60 plasma filter (LF 060-00: Infomed, Switzerland). As replacement fluids, either 5% human albumin or fresh frozen plasma was used in doses of 1–1.3 times the patient’s plasma volume. Fractionated heparin and/or citrate were used as anticoagulants. We did not use convalescent plasma for these patients.

For these patients, we monitored vital parameters: blood pressure, heart rate, oxygen saturation, ECG, temperature, and acid–base balance (pH, ionized calcium, sodium, potassium) before, during, and after the TPE session. Given the theoretical possibility of citrate accumulation, we monitored patients clinically for any potential symptoms: numbness or tingling (especially in the lips, fingers, and toes), body vibrations, metallic taste, chills, tremors, dizziness, muscle twitching, cardiac arrhythmias, etc. Laboratory and hemodynamic parameters were recorded at three time points: baseline (before TPE), mid-session (approximately 90 min), and immediately after session completion.

In Group 1, the timing of citrate anticoagulation initiation varied—some patients received it shortly after TPE started, others during the purification session, and some only toward the end of therapy. The possible side effects from citrate were analyzed in this study.

In contrast to this group of 23 patients who received citrate partway through, in Group 2, encompassing 29 sessions, citrate anticoagulation was initiated from the beginning. The reasons for starting with citrate were either irreversible clotting of the previous kit due to patient hypercoagulability, a prior session with extremely high TMP values, contraindications for heparin use, or hypertriglyceridemia with values over 2000 mg/dL.

Our study aimed to provide additional evidence supporting the safety of citrate anticoagulation in TPE. Therefore, we evaluated adverse reactions during or after the procedure. Considering that Group 2 experienced the longest exposure to citrate therapy, with or without fresh frozen plasma as replacement, any adverse effects would likely have been more pronounced than in Group 1.

From a technical standpoint, in Group 1, citrate anticoagulation in predilution was initiated when we estimated the TPE session could not be completed due to rising TMP. In cases with a rapid and consistent TMP increase, the ideal moment to initiate citrate was considered at a TMP close to 60 mmHg, although in some instances, it was initiated when TMP was already between 90 and 100 mmHg—close to or during visible circuit clotting.

The 18 mmol/L citrate solution was used in predilution at a rate of 500 to 1000 mL/h—the higher the TMP, the closer the dose was to the critical value. Since the Prismaflex device cannot eliminate more than 1000 mL/h, the citrate predilution was capped at that value. The HF404 machine cannot exceed 500 mL/h fluid loss and does not support predilution settings in TPE. In this case, we used an infusion pump connected to the kit’s port before the filter, allowing us to manually adjust citrate predilution flow rates.

Given the risk of the patient receiving more fluid than the device could remove (500 mL/h), we tried to maintain citrate predilution at a maximum of 700–800 mL/h. As the session durations were usually 3–4 h, the risk of fluid overload was estimated at 200–300 mL/h over 3–4 h (up to 1 L in total). In such cases, we administered a loop diuretic when the patient’s urine output was insufficient to maintain fluid balance. Diuresis was monitored hourly via Foley’s catheter.

All patients additionally received calcium gluconate (94 mg/mL) through an automatic syringe pump (BeneFusion Infuzomat SP3, Dutchmed, The Netherlands) at a rate of 15–25 mL/h, adjusted based on patient weight, citrate flow rate, type of replacement fluid (albumin or plasma), and serum ionized calcium levels measured during the session. Calcium gluconate administration was standardized according to patient weight (0.2–0.3 mL/kg/h). Infusion rates were subsequently adjusted every 30–60 min based on ionized calcium measurements and clinical assessment, under physician discretion.

We expected that the addition of citrate in predilution would influence treatment duration. The therapeutic plasma exchange was performed more quickly. Normally, plasma exchange is conducted at 1000–1500 mL/h, over 3–4 h in a 70 kg patient with a plasma volume of 2800 mL (40 mL/kg), replacing 1.5 times the plasma volume (4200 mL). When adding 1000 mL/h of citrate solution in predilution—requiring equivalent filtration removal—alongside a standard prescription using FFP or 5% albumin at a slightly lower rate (700–1000 mL/h), we expected the 4200 mL plasma exchange dose to be completed faster, at 1700–2500 mL/h, meaning a session duration of about 2–3 h.

Biochemical parameters were analyzed in the hospital lab using the COBAS INTEGRA 400 plus device (Roche Diagnostics, Switzerland). SARS-CoV-2 detection was performed by RT-PCR with the BIONEER extractor and EXICYCLER 96 amplifier (Bioneer, Daejeon, Republic of Korea). COVID-19 confirmation used the CFX96 Real-Time PCR system (Bio-Rad, Hercules, CA, USA). Viral RNA was extracted with the NIMBUS extractor using the STARMag 96x4 Universal Cartridge Kit (Seegene, Seoul, Republic of Korea) and amplified with the Allplex 2019-nCoV kit (Seegene, Seoul, Republic of Korea).

To evaluate the effectiveness of TPE sessions, we analyzed the main inflammatory markers before and after sessions using citrate, but only in COVID-19 patients. Since most patients had COVID-19 infection, specific autoantibodies related to autoimmune diseases were not analyzed before and after TPE.

Subgroup analyses (albumin-only vs. FFP-only replacement, COVID vs. non-COVID) were performed for exploratory purposes. Given the small sample sizes, these results should be interpreted with caution and cannot be generalized.

## 3. Statistical Analysis

The two groups were analyzed demographically and based on comorbidities and the machines used. The amount and timing of additional citrate administration were also analyzed. The evolution of key ions (Ca, K, Na, Cl), pH, lactate, hemoglobin, hemodynamic parameters, and temperature before and after sessions was studied.

The results were statistically analyzed based on the parameters and variables monitored. We compared the adverse effects observed during citrate-based TPE sessions to those seen in sessions using only heparin.

To assess the efficacy in removing inflammatory mediators, we compared pre- and post-TPE values.

Data analysis was conducted using the Statistical Package for Social Sciences v.25 (IBM SPSS Statistics, Chicago, IL, USA). A *p*-value of less than 0.05 was considered statistically significant. Paired-sample Wilcoxon tests were used due to the small sample size. This test was preferred especially in cases with extreme values for some variables (e.g., IL-6 over 5000 pg/mL). Along with *p*-values, we also report effect sizes as median differences with 95% confidence intervals to better contextualize the magnitude of change.

## 4. Results

We analyzed data from 52 TPE sessions. In Group 1, there were 23 patients with 23 TPE sessions. Group 1 included patients who started TPE with heparin and were switched to citrate at some point (Table 1). Group 2 included 14 patients who underwent 29 TPE sessions with citrate from the beginning (Table 2). Table 1 reports patient-level baseline characteristics (*n* = 23), while Table 2 reports session-level data (*n* = 29 sessions).

Table 3 shows the average and median values of measured labwork parameters before and after TPE administration, with included *p*-value for every median(see Table 3 below).

### 4.1. Results—Group 1

A total of 23 TPE sessions were performed. The moment when citrate was introduced into the treatment in predilution for regional anticoagulation varied. This was determined either by an excessive TMP alarm or by a continuously increasing TMP slope despite anticoagulation or heparin boluses. Detailed pre-/post-procedure biochemical and hemodynamic values are provided in the Supplementary Materials (Tables S1–S3).

In 7 patients, citrate was introduced within the first 20 min; in 10 patients, after the machine alarmed or due to the physician’s decision to introduce citrate occurred between 30 and 60 min; and in 6 patients, it was introduced after 90 min. The average time of introduction was 46 min at an average TMP of 82.6 mmHg. The average duration of a TPE session was 208 min (3 h and 28 min).

Other parameters obtained from the analysis of the results of the 23 sessions include a blood pump flow rate of 126 mL/min, an average amount of sodium citrate 18/0 administered per patient of 2030 mL at an average rate of 826 mL/h, and a fluid loss of 756 mL/h. Since the machines we used do not integrate citrate from the predilution into the fluid balance, patients gained a volume of 826 − 756 = 70 mL per hour.

The substitution fluids used were 5% albumin, with an average of 730 mL per patient, and FFP (fresh frozen plasma), at 1556 mL per patient. The average treatment dose of plasma exchange was 1.27 times the patient’s plasma volume (calculated as the patient’s weight multiplied by 40), which falls within the internationally recommended range of 1–1.5.

To calculate the total amount of citrate administered to the patient, we added the citrate from the sodium citrate predilution solutions and from the administered plasma. Considering that in predilution we administered 2030 mL at 18 mmol/L, we provided 36.5 mmol of sodium citrate. To this, we added 1556 mL of FFP at 20 mmol/L, which equals another 31.12 mmol of sodium citrate. Since the average session lasted 3.28 h, we administered under 10 mmol of sodium citrate per hour, which we counteracted with an average of 20 mL (4.4 mmol) of calcium gluconate per hour, representing an approximate compensation of 50%.

Next, we analyzed the values of the main ions, pH, lactate, and hemodynamic parameters before and after TPE in which 0.5% sodium citrate (18 mmol/L) was introduced during the apheresis session. Across sessions, there were no statistically significant changes in pH, lactate, sodium, potassium, chloride, hemoglobin, or hemodynamic parameters. Ionized calcium decreased modestly (1.083 → 1.032 mmol/L), without clinical manifestations (see Supplementary Table S1).

### 4.2. Results—Group 2

This group consists of patients who received 0.5% sodium citrate (18 mmol/L) in predilution from the beginning of the TPE sessions. Data from 14 patients were analyzed, and a total of 29 therapeutic plasma exchange sessions were performed. Detailed pre-/post-procedure biochemical and hemodynamic values are provided in the Supplementary Materials (Tables S1–S3).

In this group of patients, the average blood pump flow rate was 126.5 mL/min, and an average of 2206 mL of sodium citrate was used per TPE session. The rate of sodium citrate administration was 831 mL/h, which required a fluid loss managed by the machine of 771 mL/h in order to avoid volume overload in the patient. Thus, there was a net gain of 60 mL/h.

In Group 2, mean arterial pressure (TAM) decreased modestly from 79.9 to 76.8 mmHg (*p* = 0.003), and heart rate decreased from 77.6 to 75.8 bpm (*p* = 0.65).

The average treatment duration was 164 min (2 h and 24 min), which is shorter than in Group 1 (208 min or 3 h and 28 min), by nearly 1 h (54 min).

As replacement fluid, 5% human albumin was used, with an average of 827 mL per patient, and fresh frozen plasma (FFP) at 1106 mL per patient. If we also add the citrate used in predilution, we can quantify the total substitution fluid used as replacement at 4175 mL per patient. This value represents 1.26 times the patient’s plasma volume (calculated as 40 mL × body weight), which corresponds to an ideal treatment dose.

To counteract the systemic effects of citrate, used as regional anticoagulation, and considering the additional citrate present in the administered blood products (FFP), calcium gluconate 94 mg/mL was administered at an average rate of 24 mL/h.

Serum calcium values before and after the TPE session, along with the main monitored ions, are shown in Supplementary Table S2. This table also displays the main hemodynamic values, pH, lactate, and temperature. Ionized calcium decreased significantly (1.10 → 1.04 mmol/L; *p* = 0.0003). Modest reductions were observed in sodium (138.9 → 137.3 mmol/L; *p* < 0.001), chloride (105.2 → 104.4 mmol/L; *p* = 0.03), potassium (3.65 → 3.49 mmol/L; *p* = 0.0006), and mean arterial pressure (79.9 → 76.8 mmHg; *p* = 0.003); other parameters remained stable (see Supplementary Table S2).

In Group 2, where we used predilution treatment with citrate, we compared the results of the same parameters in patients who received citrate-only replacement fluid with those who received heparin-only anticoagulation. This comparison illustrates the greater or lesser impact in situations where there is theoretically an additional citrate load in the replacement fluid.

There were six treatments in which only albumin was used as a replacement fluid, with an average blood pump flow rate of 130 mL/min, an average treatment duration of 151 min, and an average exchange dose of 1.22× the patient’s plasma volume. A total of 3683 mL of replacement fluid was used, of which 2016 mL was citrate in the pre-filter and 1667 mL was albumin in the post-filter.

There were also six treatments in which only FFP (fresh frozen plasma) was used as a replacement fluid, with an average blood pump flow rate of 125 mL/min, an average treatment duration of 158 min, and an average exchange dose of 1.12× the patient’s plasma volume. A total of 3780 mL of replacement fluid was used, of which 2300 mL was citrate in the pre-filter and 1440 mL was FFP in the post-filter. In citrate-treated sessions using albumin only versus FFP only, trends in electrolytes and hemodynamics were comparable; the FFP-only subgroup showed a slightly greater iCa decline, consistent with the additional citrate in plasma products (see Supplementary Table S3).

The results show that TPE with citrate in these patients was well tolerated in terms of the parameters monitored above. The only difference observed, which was statistically significant but not clinically relevant, was in ionized calcium levels, which were slightly lower in patients who received only FFP compared to those who received only albumin.

Since there were 32 patients in these groups with COVID-related pathology, we also monitored the evolution of key inflammatory markers before and after the TPE sessions in which 0.5% sodium citrate was introduced either during the session or from the beginning as predilution anticoagulation.

The results demonstrate the effectiveness of the TPE sessions performed with citrate, also in terms of the statistically significant reduction in inflammatory markers, including IL-6, ferritin, D-dimers, CRP, LDH, fibrinogen, and ESR (erythrocyte sedimentation rate).

To further support the efficacy of these citrate-based TPE sessions, we also analyzed the evolution of triglyceride levels before and after the procedures (Table 4).

To evaluate both the safety and effectiveness of regional citrate anticoagulation in therapeutic plasma exchange (TPE), we performed a comparative statistical analysis between the two patient groups. The comparative descriptive analysis of the two groups is shown in Table 5.

These results suggest that both groups were demographically similar, with comparable patient profiles. TPE duration was significantly shorter in Group 2 (*p* < 0.05), demonstrating increased efficiency when citrate was used from the beginning.

There were four mild allergic reactions during plasma administration (manifested as urticaria), all resolved with antihistamines. No reactions were related to citrate.

Ionized calcium levels, both before and after TPE, did not differ significantly between groups, indicating comparable safety of citrate use. Moreover, citrate dosage and administration rates were not significantly different, suggesting consistent application protocols in both groups.

To test for statistically significant differences between groups, we performed independent *t*-tests for variables with approximately normal distributions and Mann–Whitney U tests for variables with non-normal distributions or unequal variances

The outcomes of the statistical tests are summarized below (Table 6).

The only statistically significant difference observed between the groups was in the TPE session duration, which was significantly shorter in patients who received citrate from the beginning (Group 2). This suggests that initiating citrate anticoagulation early may optimize session efficiency, likely due to better flow stability and fewer clotting interruptions.

**Figure 3:**
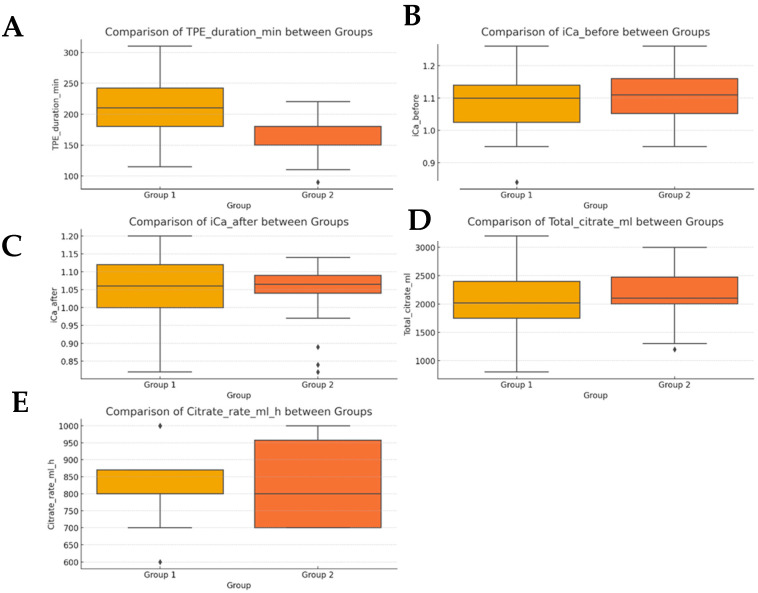
Box plot for key variables. (**A**) comparison of TPE duration (in minutes) between groups. (**B**) Comparison of ionized calcium levels between groups, before infusion of citrate. (**C**) Comparison of ionized calcium levels between groups, after the infusion of citrate. (**D**) Comparison of total citrate administered (in mL) between groups. (**E**) Comparison of citrate administration rate (in mL/h) between groups.

To complement the numerical analysis, box plots were generated for each key variable (see Figure 3).

These clearly illustrate the distribution and spread of values in both groups. Notably, TPE duration shows a markedly lower median and narrower distribution in Group 2. Ionized calcium levels before and after treatment remain consistent across groups. Citrate administration volumes and rates are similar, supporting protocol consistency.

These findings reinforce the feasibility and safety of citrate use in TPE, both as a primary anticoagulant and as a rescue option when heparin fails. The absence of significant ionized calcium drops suggests that citrate toxicity was well controlled, likely due to careful calcium gluconate compensation during sessions. Additionally, the efficiency gains (shorter session times) observed in Group 2 may have implications for resource allocation, cost-effectiveness, and workflow optimization in intensive care settings.

The statistical analysis therefore supports the integration of citrate-based anticoagulation into standard TPE protocols, provided that patient monitoring and fluid balance are properly managed.

Detailed variable tables are presented in the Supplementary Materials to streamline the main results.

## 5. Discussion

Our study aimed to evaluate the use of sodium citrate as regional anticoagulation, added via predilution in machines that perform therapeutic plasma exchange (TPE) through filtration. The rate of citrate administration received by patients was chosen arbitrarily. This rate was lower than the widely recommended and used dose for patients undergoing hemofiltration, which is 3 mmol/L of blood. In our study, the average rate used was below 2 mmol/L. As for the calcium supplementation, our protocol is consistent with Krammer et al. (0.19 mmol/min Ca^2+^) [15]. The current study highlights the use of dynamic citrate introduction based on TMP trends, in comparison with other studies that rely on a fixed citrate anticoagulation protocols [16,17]. Our approach to a guided switch from heparin to citrate is a novel, safety-oriented tactic that may be particularly relevant in patients with a high prothrombotic state, as well as those with COVID-19. The COVID-19 pandemic led to a large number of changes in healthcare systems. Understanding the course of the disease was a major challenge, and to date a significant amount of research has been undertaken to explore different aspects and outcomes of it [18,19]. COVID-19 patients are vulnerable both in the acute phase and in the post-COVID syndrome, leading to high mortality rates due to the prothrombotic state and the severe imbalance in the inflammation cascade that leads to post-COVID-19 pulmonary or cardiac complications [20].

Given that the average blood pump flow rate was between 120 and 130 mL/min, we can state that our patients received a much lower amount of citrate compared to the study by Sebastien Kislling et al. [21], who used flow rates of 200 mL/min, resulting in a higher citrate dose. From this perspective, we developed a model in which a lower dose of citrate (under 50%) is used, which was compensated for with calcium at approximately 50%, significantly reducing the risk of citrate toxicity, as measured by the plasma calcium/ionized calcium ratio.

These assumptions were confirmed by our results. There were no adverse reactions related to citrate administration during any of the TPE sessions using citrate. The only adverse events observed were four allergic reactions to plasma administration (7.69%).

The biochemical changes observed post-TPE were mild and clinically well tolerated. Ionized calcium (iCa) decreased modestly in both groups (Group 1: 1.083→1.032 mmol/L; Group 2: 1.10→1.04 mmol/L), in line with findings by Jiao et al., who reported post-session iCa values of 1.03 mmol/L [22]. No cases of symptomatic hypocalcemia or citrate toxicity were documented.

Other parameters such as pH, lactate, sodium, and potassium remained stable. A minor increase in pH was observed in Group 2 (from 7.407 to 7.412), supporting the known alkalinizing effect of citrate metabolism, as previously described by Kramer L et al. [15]. Hemodynamic parameters, including mean arterial pressure and heart rate, showed no significant post-TPE variation, corroborating the safety of RCA in critically ill populations [16,17].

**Relation to prior work:** Our TMP-guided, dynamic use of RCA differs from fixed-dose approaches reported for TPE or DFPP, yet the observed iCa trajectories and overall metabolic stability align with prior safety reports. The shorter session duration with early RCA is consistent with the concept that preventing micro-clotting preserves flow stability, echoing efficiency signals from models such as the one proposed by Kissling et al. and segmental RCA in DFPP. Notably, our filtration-based TPE workflow on devices not natively designed for citrate required fluid-balance coupling, which may explain small differences from centrifugation-based platforms.

**Potential confounding and bias:** Group allocation was non-random (heparin-first vs. RCA-first), and citrate timing varied in Group 1 (mid-session switch), which could attenuate biochemical effects relative to Group 2. Device caps (Prismaflex ≤ 1000 mL/h loss; HF404 ≤ 500 mL/h), replacement fluid choice (FFP vs. albumin), and COVID-19 disease severity may each influence iCa and hemodynamics. We did not perform multivariable adjustment, so residual confounding is possible; future randomized or propensity-matched designs are warranted.

The citrate present in FFP (fresh frozen plasma) provided by blood centers does not seem to have the same impact on calcium homeostasis as the same amount of citrate administered in predilution bags to the plasma filter. This may be due to the composition of FFP, which also contains calcium and other coagulation factors [23].

Our study suggests that regional anticoagulation with sodium citrate in TPE sessions should follow blood pump flow rates similar to those in hemofiltration [24], where the flows are lower than in TPE and citrate anticoagulation is performed at lower doses to prevent toxicity.

The amount of citrate administered in a single TPE session—accounting for both citrate used as an anticoagulant and that from FFP—is approximately 10 mmol/h in our study, with a maximum of 48 mmol/h when both maximum citrate predilution (1000 mL/h) and FFP replacement fluid (1500 mL/h) were used. In a classic CRRT (Continuous Renal Replacement Therapy) session with citrate, about 1200 mL of citrate per hour is used, which equates to 480 mmol per day. Therefore, in 3 h of TPE with citrate, an average of 30 mmol of citrate was used, with a maximum possible dose of 144 mmol. These 144 mmol used during a 3 h TPE session would only correspond to 7 h of classic CRRT with citrate. Given that CRRT with citrate typically lasts 2–3 days, the risk of toxicity in TPE with citrate, even at maximal doses, is significantly lower.

Using citrate in predilution requires that the machines used for TPE sessions generate fluid loss to avoid volume overload. Therefore, the total replacement fluid volume used must include predilution citrate (800–1000 mL) and the post-filter fluid (1000–1500 mL). This setup leads to an increase in treatment speed and a shorter treatment duration. For example, for a patient receiving 5000 mL of replacement fluid, instead of 1500 mL/h, the rate becomes 2500 mL/h, reducing the treatment time from 3 h and 20 min to 2 h—a reduction of 1 h and 20 min.

In our study, the treatment duration in Group 2 (where citrate was introduced from the beginning) was 54 min shorter than in Group 1 (where citrate was introduced during the session).

The main parameters monitored before and after TPE with citrate, whether introduced at the beginning or during the session, included blood pressure, heart rate, oxygen saturation, ECG, temperature, and acid–base balance (pH, ionized calcium, sodium, potassium). These values did not change significantly in our study (see Table 2 and Table 4), confirming the safety of this procedure. This is further supported by Table 5, which analyzed the difference in ionized calcium before and after TPE with citrate in patients receiving FFP versus 5% human albumin as replacement fluid. The ionized calcium level was only slightly lower at the end of the session (1.02 vs. 1.10 mg/dL).

Citrate anticoagulation does not reduce the efficacy of TPE sessions. The evaluation of inflammatory markers and triglyceride levels before and after TPE confirms this (see Supplementary Files: Tables S2 and S3). When comparing albumin-only vs. FFP-only replacement within Group 2, sessions with FFP were associated with a slightly greater iCa decline, consistent with the additional citrate content in plasma products (approximately 20 mmol/L) [9]. However, both subgroups maintained acceptable metabolic parameters without clinical sequelae. These findings mirror those of Betz et al. [9], who observed increased citrate load and calcium requirement with FFP.

One of the most striking findings of this study is the significant reduction in inflammatory markers in patients undergoing citrate-based TPE, particularly in the COVID-19 subgroup (*n* = 32). Interleukin-6, ferritin, D-dimers, CRP, LDH, and fibrinogen all showed statistically and clinically significant reductions post-procedure (all *p* < 0.001). Although statistically significant, these changes remained within clinically acceptable ranges and did not cause symptoms. The predictable decrease in ionized calcium was effectively counteracted with calcium supplementation. Therefore, while the findings highlight the need for monitoring, they do not indicate clinically harmful effects, supporting the safety of citrate anticoagulation in this setting. These data support the hypothesis that TPE, when combined with RCA, can effectively attenuate the hyperinflammatory response, aligning with findings by Bouayed et al. [25] and Connelly-Smith et al. [2].

In addition, in the subgroup of patients with hypertriglyceridemia (*n* = 4), triglyceride levels decreased by over 70% post-TPE (3680 → 969 mg/dL, *p* < 0.005), demonstrating the efficacy of citrate-based apheresis in metabolic emergencies.

It is important to note that none of the same parameters showed statistically significant changes in Group 1 when compared to Gorup 2. This may be due to shorter exposure to citrate, variability in when citrate was added, or fewer complete paired samples. This could be explained by the fact that citrate anticoagulation has measurable biochemical effects. Group 2, where citrate was used from the beginning, showed statistically significant reductions in iCa, Na, Cl, K, and hemoglobin. These are all expected and manageable effects during TPE with citrate, due to calcium binding by citrate and dilution effects from fluid shifts and replacement solutions.

Despite these significant changes, the values remained clinically acceptable. There were no adverse events reported related to electrolyte imbalance, and calcium was properly compensated for with calcium gluconate infusion.

The absence of significant changes in Group 1 suggests that shorter or later citrate exposure (added mid-session) may reduce the physiological impact. This supports the controlled and safe use of citrate, even when used reactively.

**Clinical implications.** Our data support regional citrate as (i) a first-line anticoagulant in patients with heparin contraindications or high bleeding risk and (ii) a rescue strategy when rising TMP under heparin signals imminent filter clotting. A pragmatic algorithm is to start with RCA in bleeding-prone or hypercoagulable patients and to switch dynamically from heparin to RCA when TMP rises despite boluses. This dual role may help prevent treatment failure, conserve kits, and shorten session duration.

## 6. Limitations and Future Directions

This prospective observational work lacks randomization and long-term follow-up; we did not capture post-ICU outcomes (e.g., delayed hypocalcemia, rehospitalization) or filter life comparisons. Selection bias is possible (e.g., TMP-guided crossover decisions), and timing variability of RCA initiation may influence biochemical deltas. Generalizability is limited by local protocols and device constraints. Therefore, standardized, multicenter trials with long-term outcomes are needed to confirm durability and external validity.

Potential confounders in our study include heterogeneity of underlying conditions (COVID-19, myasthenia gravis, hypertriglyceridemia), variability in replacement fluids (albumin vs. FFP), and differences in timing of citrate initiation. These factors may have influenced biochemical results or session duration. Calcium supplementation protocols may also have acted as effect modifiers by mitigating citrate’s systemic effects. Although our analysis attempted to account for these, residual confounding cannot be excluded.

This study is limited by its relatively small sample size in some subgroups (e.g., albumin-only or FFP-only arms). Beyond the small subgroup sizes, this was a single-region, two-ICU experience with 26 patients/52 sessions, which limits external validity. Device platforms (Prismaflex vs. HF404), replacement fluids, and local workflows may differ elsewhere. Future multicenter studies with larger and more heterogeneous cohorts are required to validate effectiveness and safety across diverse case-mixes and operational settings. The TMP-guided transition strategy, while effective, requires prospective validation in larger cohorts. Future studies should also explore long-term outcomes, citrate accumulation markers, and personalized calcium replacement protocols based on real-time iCa monitoring.

We were not powered to analyze patients with marked baseline calcium derangements or conditions associated with impaired citrate metabolism (e.g., severe hepatic dysfunction, shock) as dedicated subgroups. Such phenotypes were either rare or clinically heterogeneous in our cohort and merit a priori stratification in future trials.

## 7. Conclusions

This study demonstrates that therapeutic plasma exchange (TPE) using regional citrate anticoagulation is not only safe but also effective when applied either from the beginning of the session or introduced during the session in response to high transmembrane pressures indicating imminent clotting.

In patients where heparin was insufficient or contraindicated, the addition of citrate successfully salvaged TPE sessions, preserved equipment, and avoided treatment interruptions. In Group 1 (heparin first, citrate added), 21 of 23 sessions were completed after citrate was introduced, confirming its value as a rescue anticoagulant strategy. These findings show RCA is a viable alternative when heparin is contraindicated and as a complementary rescue when clotting risk escalates during TPE.

When citrate was used from the outset (Group 2), sessions were significantly shorter, with a reduction of nearly one hour per session. This has direct implications for ICU efficiency, staff workload, and resource optimization, especially in high-demand settings like the COVID-19 pandemic or in patients with autoimmune disorders requiring multiple TPE sessions.

Statistical analysis confirmed that although some biochemical parameters shifted significantly (e.g., ionized calcium, sodium, chloride, potassium), these changes remained within clinically acceptable ranges and were predictable and manageable with standard calcium supplementation protocols. No clinically significant adverse events related to citrate toxicity were observed in either group. These findings support the conclusion that regional citrate anticoagulation in TPE is a viable and potentially superior alternative to heparin, particularly when hypercoagulability or filter clotting is anticipated. The ability to reduce session duration, improve treatment reliability, and maintain patient safety makes citrate an attractive first-line or backup option.

Therefore, we see fit to recommend that TPE machines should integrate software options to support citrate-based protocols, including automatic citrate flow control and fluid balancing. Moreover, ICU teams should receive training on the safe use of citrate, including monitoring and compensatory calcium infusion. Lastly, further multicenter trials are warranted to validate these findings across different clinical environments and patient populations.

## Figures and Tables

**Table 1:** Demographic and baseline characteristics of patients who received citrate during TPE (*n* = 23).

Age-mean	49.39 (±12.63)
Weight	86.8 (±16.92)
Gender	
Male	14
Female	9
Comorbidities	
COVID-19	19
Myasthenia Gravis	1
Polyradiculonevritis	2
Hypertriglyceridemia	1
Prismaflex with TPE 2000 plasma filter	15
HF 440 with Granopen 60 plasma filter	8

**Table 2:** Demographic and baseline characteristics of patients who received citrate from the beginning of TPE (*n* = 29).

Age-Mean	48.3
Weight	80.3
Gender	
Male	16
Female	13
Comorbidities	
COVID-19	13
Myasthenia Gravis	3
Polyradiculonevritis	10
Hypertriglyceridemia	3
Prismaflex with TPE 2000 plasma filter	18
HF 440 with Granopen 60 plasma filter	11

**Table 3:** Average and median values before and after TPE, *p*-value (*n* = 33).

Variable	Average/Median Before TPE	Average/Median After TPE	*p*-Value for Median
IL-6, pg/mL	799/129	480/79	0.0003
Ferritin, µg/L	2364/1529	1660/1120	<0.0001
D-dimers, µg/mL	5.18/1.9	3.50/1.57	0.0024
CRP, mg/L	122/88	87/60	0.0001
LDH, U/L	579/512	461/419	<0.0001
PCT, ng/mL	2.28/0.33	2.26/0.42	0.34
Fibrinogen, g/L	4.90/4.23	3.42/3.26	<0.0001
ESR, mm/h	46/35	22/15	<0.0001
Leucocytes, ×10^3^/µL	14/13	16/15.5	0.19
% Lymphocytes	7.66/5.2	8.14/5.5	0.53
Lymph abs, ×10^3^/µL	0.96/0.69	1.11/0.80	0.003
TAM, mmHg	80.9/77	81.9/80	0.9
Temperature, °C	36.5/36.4	36.6/36.4	0.26
BUN, mg/dL	72.7/58.5	74.2/62	0.29
Creatinine, mg/dL	1.02/0.8	1.08/0.81	0.98

(IL-6: Interleukin 6; CRP: C-reactive protein; LDH: lactate dehydrogenase; PCT: Procalcitonin; ESR: erythrocyte sedimentation rate; TAM: mean arterial pressure; BUN: blood urea nitrogen).

**Table 4:** Average and median values before and after TPE, *p*-value (*n* = 4).

Variable	Average/Median Before TPE	Average/Median After TPE	*p*-Value for Median
TRIGLICERIDE	3680 mg/dL	969 mg/dL	*p* < 0.005

**Table 5:** Comparative analysis of Group 1 and Group 2.

Parameter	Group 1 (Mean ± SD)	Group 2 (Mean ± SD)
Age (years)	49.4 ± 12.6	48.3 ± 12.1
Weight (kg)	86.8 ± 16.9	80.3 ± 12.1
TPE duration (min)	208.0 ± 5	164.0 ± 7.3
Ionized calcium (before)	1.09 ± 0.10 mmol/L	1.11 ± 0.08 mmol/L
Ionized calcium (after)	1.05 ± 0.10 mmol/L	1.04 ± 0.08 mmol/L
Total citrate (mL)	2030 ± 21.3	2206 ± 18.6
Citrate rate (mL/h)	826 ± 15.3	831 ± 17.4

**Table 6:** Statistically significant differences between the two groups.

Variable	Mann–Whitney U *p*-Value	*T*-Test *p*-Value
TPE duration (min)	<0.001	<0.001
Ionized Ca (Before)	0.220	0.188
Ionized Ca (After)	0.737	0.754
Total citrate (mL)	0.121	0.140
Citrate rate (mL/h)	0.942	0.870

## Data Availability

Data will be made available on a valid written request to the corresponding authors.

## Supplementary Materials

The following supporting information can be downloaded at: https://www.mdpi.com/article/10.3390/clinpract15100172/s1, Table S1. Main values studied before and after TPE with citrate introduced during the apheresis session. Table S2. Main values studied before and after TPE with citrate introduced from the beginning of the apheresis session. Table S3. Main values studied before and after TPE with citrate in patients where only 5% human albumin or only fresh frozen plasma was used as replacement fluid. Standardized protocol for regional citrate anticoagulation (RCA) in TPE.
