# Supplementary material for: Safety and Efficacy of Citrate Anticoagulation in Therapeutic Plasma Exchange: A Clinical Study
Source: Clin Pract. 2025 Sep 23;15(10):172. doi: 10.3390/clinpract15100172 (PMC12563097; doi:10.3390/clinpract15100172)
Supplement: Supplementary file 1 [file clinpract-15-00172-s001.zip › clinpract-3837867-supplementary.pdf]

Supplementary materials

Article

Safety and Efficacy of Citrate Anticoagulation in Therapeutic Plasma Exchange: A Clinical Study

Ciprian Gîndac [1,2,†], Tamara Mirela Poroșnicu [1,3,†], Nilima Rajpal Kundnani [4,5,*], Nicoleta Sgăvârdea[3,6,*], Claudiu Rafael Bârsac [1,2,6], Vlad Meche [6], Adelina Băloi [1,2,6], Laura Alexandra Nussbaum [7], Ovidiu Horea Bedreag [1,2], Dorel Săndesc [1,2] and Păpurică Marius [1,2]

[1] Department of Anaesthesia and Intensive Care, "Victor Babes"University of Medicine and Pharmacy, 300041, Timisoara, Romania
[2] Clinic of Anesthesia and Intensive Care, "Pius Brinzeu" Emergency Clinical County Hospital, 300723 Timisoara, Romania
[3] Compartment of Anesthesia and Intensive Care, "Dr. Victor Babes"Infectious Diseases and Pulmonology Clinical Hospital, 300310 Timisoara, Romania
[4] University Clinic of Internal Medicine and Ambulatory Care, Prevention and Cardiovascular Recovery, Department VI-Cardiology, "Victor Babes" University of Medicine and Pharmacy, 300041 Timisoara, Romania
[5] Research Centre of Timisoara Institute of Cardiovascular Diseases, "Victor Babes" University of Medicine and Pharmacy, 300041 Timisoara, Romania
[6] Doctoral School, "Victor Babes" University of Medicine and Pharmacy, 300041 Timisoara, Romania
[7] Department of Pedopsychiatry, "Victor Babes" University of Medicine and Pharmacy, 300041 Timisoara, Romania
* Correspondence: knilima@umft.ro (N.R.K.); nicoleta.cotaia@umft.ro (N.S.)
† These authors contributed equally to this work.

Table S1. Main values studied before and after TPE with citrate introduced during the apheresis session.

		Before TPE	After TPE	Statistical significance (p value)
1	Ph	7.40	7.40	0.903
2	Lactate mmol/l	2.27	2.17	0.702
3	iCa mmol/l	1.083	1.032	0.198
4	Na mmol/l	138.3	136.2	0.111
5	Cl mmol/l	103.3	102.6	0.130
6	K mmol/l	3.68	3.58	0.373
7	Hb g/dl	12.80	12.79	0.409
8	TAM mmHg	81.13	78.96	0.104
9	AV /min	78.56	77	0.822
10	Temperature	36.5	36.6	0.566

Table S2. Main values studied before and after TPE with citrate introduced from the beginning of the apheresis session.

		**Befor TPE**	**After TPE**	**Statistical significance (p value)**
1	Ph	7.407	7.412	0.06
2	Lactate mmol/l	1.90	2.02	0.24
**3**	**iCa mmol/l**	**1.10**	**1.04**	0.0003
4	Na mmol/l	138.9	137.3	<0.001
5	Cl mmol/l	105.2	104.4	0.03
6	K mmol/l	3.65	3.49	0.0006
7	Hb g/dl	12.80	12.79	<0.001
8	TAM mmHg	79.89	76.82	0.003
9	AV /min	77.55	75.82	0.65
10	Temperature	36.87	36.63	0.004

Table S3. Main values studied before and after TPE with citrate in patients where only 5% human albumin or only fresh frozen plasma was used as replacement fluid.

		Albumin only 5% n=6		PPC only n=6	
		Before TPE	After TPE	Before TPE	After TPE
**1**	Ph	7,37	7,40	7,39	7,40
2	Lactate mmol/l	2,61	2,74	1,73	1,81
**3**	**iCa mmol/l**	**1,12**	**1,10**	**1,07**	**1,02**
4	Na mmol/l	141	139,2	141	139,5
5	Cl mmol/l	105,6	104	105	105
6	K mmol/l	3,68	3,54	3,78	3,62
7	Hb g/dl	11.1	10,8	12,5	12,2
8	TAM mmHg	72,1	70,8	92,6	89,5
9	AV /min	78,1	75,8	88,9	83,1
10	Temperature	36,7	36,4	36,8	36,6

Standardized protocol for regional citrate anticoagulation (RCA) in TPE

Initiation triggers (any of the following):

- continuously rising TMP with slope suggestive of imminent clotting;
- TMP ≥ 60–100 mmHg with an upward trend or “filter clogged” alarm;
- heparin contraindication or high bleeding risk;
- prior rapid filter clotting;
- severe hypertriglyceridemia (>2000 mg/dL).

Citrate solution and starting rate: 0.5% sodium citrate (18 mmol/L) infused pre-filter targeting **~2–3 mmol citrate per liter of blood** entering the filter. With typical blood flows of 120–130 mL/min, this corresponded to **500–800 mL/h** (upper cap **1000 mL/h** on Prismaflex; HF404 via external infusion pump). The exact start within this range was selected according to TMP severity.

Fluid balance coupling: Device “fluid loss” was set to **80–100%** of the pre-filter citrate rate to limit net balance to **≤ +60 mL/h**; remaining replacement was delivered as albumin and/or FFP.

Calcium supplementation (systemic): Calcium gluconate 94 mg/mL at **0.2–0.3 mL/kg/h** baseline; titrate **every 30–60 min** to maintain **systemic iCa 1.00–1.20 mmol/L**.

- **if iCa < 0.95 mmol/L or symptoms**, increase calcium by **+5–10 mL/h** and/or reduce citrate by **100–200 mL/h**;
- **if iCa < 0.90 mmol/L**, pause citrate until iCa recovers ≥ 1.00 mmol/L, then resume at a lower rate.

Laboratory and clinical monitoring: iCa, Na^+, K^+, Cl^-, pH, lactate, hemodynamics **at baseline, ~90 min, and end-session**; continuous ECG and non-invasive blood pressure; clinical monitoring for citrate effects (perioral/finger paresthesia, tremor, chills, arrhythmia).

Special situations: With FFP replacement (citrate load ~20 mmol/L), anticipate higher calcium requirements; prefer the lower end of citrate rates when TMP allows. For HF404 (no built-in predilution), use infusion pump pre-filter and strictly couple fluid loss to the infusion to avoid fluid overload.

De-escalation/stop rules: Reduce or stop citrate if TMP normalizes and remains stable < 60 mmHg for ≥ 30 min, or if persistent iCa instability occurs despite supplementation.

Targets: uninterrupted session completion; avoidance of symptomatic hypocalcemia or alkalosis; session duration optimization.
